# Monocytes Derived From Human Pluripotent Stem Cells Engineered for Detection of Pyrogens

**DOI:** 10.1111/cpr.70233

**Published:** 2026-05-21

**Authors:** Jingjing Liu, Bingyu Cai, Tingting Gao, Wumei Wei, Liu Wang, Qi Zhou, Wei Li, Jie Hao, Jun Wu

**Affiliations:** ^1^ State Key Laboratory of Organ Regeneration and Reconstruction Institute of Zoology, Chinese Academy of Sciences Beijing China; ^2^ University of Chinese Academy of Sciences Beijing China; ^3^ Bejing Institute for Stem Cell and Regenerative Medicine Beijing China; ^4^ National Stem Cell Resource Center Institute of Zoology, Chinese Academy of Sciences Beijing China

**Keywords:** human embryonic stem cells, luciferase, monocytes, NF‐κB reporter system, pyrogens

## Abstract

Pyrogens, including endotoxins and non‐endotoxins, are key factors that impact the safety of parenterally administered drugs as contaminants. Currently, traditional pyrogen detection methods primarily rely on the Rabbit Pyrogen Test (RPT) and the Limulus Amebocyte Lysate (LAL) assay, both of which are derived from animal‐based systems. However, both methods possess certain limitations. The RPT demonstrates lower reproducibility and a higher false‐positive rate compared to the LAL assay; in contrast, the LAL test is limited to detecting endotoxins from Gram‐negative organisms. The Monocyte Activation Test (MAT), which is based on monocytes, has emerged as the most promising alternative to traditional pyrogen detection methods. However, the MAT is operationally complex, time‐consuming, and exhibits significant individual variability; it typically requires a substantial volume of human blood, thereby considerably limiting its practical application. To overcome these limitations, we developed a rapid pyrogen detection method based on monocytes derived from human embryonic stem cells (hESCs), integrated with a luciferase reporter system. By leveraging the stability and pluripotency of hESCs, we can obtain numerous hESC‐derived monocytes (hESC‐Mono) through directed in vitro differentiation. These hESC‐Mono exhibit batch‐to‐batch consistency and closely resemble peripheral blood monocytes in functionality. We have demonstrated that hESC‐Mono possess sensitive reporting capabilities for at least three types of pyrogens: lipopolysaccharide (LPS), a major component of the cell wall of Gram‐negative bacteria; lipoteichoic acid (LTA), a key constituent of the cell wall of Gram‐positive bacteria; and fungal Zymosan. Furthermore, we have confirmed that hESC‐Mono maintain stable expression of Toll‐like receptors, which are among the primary determinants of the reliable detection of pyrogens. Consequently, we have successfully developed and validated a novel pyrogen detection method based on the integration of an in vitro hPSC‐directed differentiation system with a luciferase‐based reporter assay, thereby offering a promising alternative to traditional pyrogen detection methods.

## Graphical abstract

In summary, we have developed a monocyte‐based reporter system using hESC‐Mono for interrogating the NF‐κB signalling axis, a central pathway governing pyrogenic responses. Our platform enables rapid and dose‐dependent detection of both endotoxins (LPS) and non‐endotoxins (LTA and Zymosan).

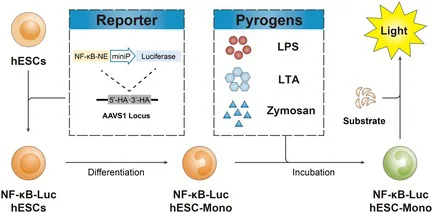

## 1 Introduction

Pyrogens, including endotoxins and non‐endotoxin pyrogens, represent common contaminants in parenteral pharmaceuticals (e.g., biologics, chemical drugs, traditional Chinese medicine injections) and medical devices [1, 2]. These contaminants are capable of inducing febrile reactions and, in severe cases, may lead to life‐threatening multi‐organ failure in patients [3]. Furthermore, the synergistic effects of coexisting pyrogens can significantly amplify their toxicity [4]. Consequently, pyrogen testing is a pharmacopoeia‐mandated requirement to ensure the safety of injectable therapeutics.

However, as pharmacopeial‐recognized traditional pyrogen detection methods, the Rabbit Pyrogen Test (RPT) and Limulus Amebocyte Lysate (LAL) assay face significant limitations [2, 5, 6]. The RPT necessitates extensive use of laboratory animals, incurs high operational costs and demonstrates high inter‐experimental variability due to species‐specific differences between rabbits and humans [7, 8]. Moreover, it provides only qualitative pass/fail results, thereby limiting its utility for quantitative safety assessments. Consequently, the RPT has been withdrawn from the European Pharmacopoeia as of the 1 July 2025 revision (Ph. Eur. 5.1.13). The LAL assay, while specific for Gram‐negative bacterial endotoxins, is incapable of detecting non‐endotoxin pyrogens and is susceptible to interference from β‐glucans, serine proteases, or detergents [9, 10]. Although β‐glucan‐induced interference in the LAL assay can be mitigated by inactivating Factor G or incorporating β‐glucan inhibitors [11], the challenge of endotoxin masking caused by detergents remains unresolved. Furthermore, the declining populations of horseshoe crab pose a threat to the long‐term sustainability of LAL reagent production [7, 12]. Similarly, the recombinant Factor C (rFC) assay, a derivative of LAL, although superior to the RPT in endotoxin detection sensitivity and increasingly adopted in various applications [13, 14, 15], shares the same limitation as LAL in detecting only endotoxins. Given the inherent limitations of traditional pyrogen detection methods, including the RPT and LAL assays, there is an urgent need to develop robust in vitro alternatives. Indeed, the regulatory guidelines of the European Pharmacopoeia now allow the waiver of compendial RPT or LAL testing for commercial biologics if validated in vitro methods demonstrate equivalent or superior reliability (Ph. Eur. 5.1.6) [8].

Fever, a hallmark symptom of infections mediated via Toll‐like receptors (TLRs), is induced by exogenous pyrogens such as lipopolysaccharide (LPS; derived from Gram‐negative bacteria), lipoteichoic acid (LTA; associated with Gram‐positive bacteria) and fungal Zymosan [16, 17]. Notably, these pyrogens are not restricted to live microorganisms. TLRs, which are abundantly expressed on monocytes, facilitate the recognition of diverse pathogen‐associated molecular patterns (PAMPs), thereby initiating inflammatory signalling cascades that culminate in the secretion of pyrogenic cytokines, including interleukin‐6 (IL6), interleukin‐8 (IL8), interleukin‐1β (IL1β) and tumour necrosis factor‐α (TNFα), and subsequent febrile responses. Leveraging this mechanism, advanced in vitro pyrogen tests, such as the Monocyte Activation Test (MAT), have been developed [18, 19]. MAT quantifies the production of cytokines (IL‐6, IL‐8, IL‐1β and TNF‐α) in human monocyte cultures, providing a physiologically relevant, animal‐free platform for broad‐spectrum pyrogen detection [20, 21].

Currently, peripheral‐blood mononuclear cells (PBMCs)—which include not only CD14^+^ monocytes but also T, B and NK lymphoid subsets—are the primary cell source used in the MAT. Compared to RPT and LAL assay, the MAT demonstrates significantly improved reproducibility and reliability, along with the unique ability to detect non‐endotoxin pyrogens [22, 23, 24]. Validated by the European Centre for the Validation of Alternative Methods (ECVAM) and formally adopted by the European Pharmacopoeia since 2010, the MAT has since been incorporated into the Chinese Pharmacopoeia, Indian Pharmacopoeia and International standards, including ISO 10993‐11 and ISO/TR 21582:2021. MAT utilizes human‐derived cells to more accurately recapitulate human immune responses compared to species‐specific RPT [7]. Furthermore, MAT enables quantitative endotoxin detection without reliance on animal use. However, its practical utility is limited by challenges in monocyte sourcing. Primary monocytes derived from human PBMCs exhibit batch‐to‐batch variability and donor‐dependent responses, while immortalized cell lines, such as THP‐1 and Mono Mac 6, display altered phenotypes that deviate from physiological monocyte behaviour [25, 26, 27, 28]. Additionally, genetic modification of primary monocytes using non‐viral methods remains inefficient, and MAT workflows are time‐consuming (> 24 h for cytokine‐based ELISA readouts) [21]. Engineered cell lines, such as HEK293 or A549, expressing TLRs and co‐factors have been explored but suffer from insufficient sensitivity [29, 30, 31, 32]. These limitations highlight the necessity for innovative strategies to optimize MAT protocols and enhance detection efficiency.

NF‐κB activation represents a critical mechanism underlying pyrogen‐induced fever, as the interaction of PAMPs with TLRs on immune cells triggers intracellular signalling cascades that rely on NF‐κB pathway activation [33, 34]. This signalling axis promotes the transcriptional upregulation and secretion of pro‐inflammatory cytokines, including IL6, IL8, IL1β and TNFα, which mediate febrile responses. The emergence of luciferase reporter systems has facilitated the development of rapid pyrogen detection platforms by enabling the quantitative monitoring of NF‐κB‐responsive transcriptional activity [35].

Meanwhile, human pluripotent stem cells (hPSCs), including human embryonic stem cells (hESCs) and induced pluripotent stem cells (iPSCs), exhibit two transformative capabilities: the capacity for unlimited scalability in mass production coupled with the ability to complete crucial QC before use, and the potential to differentiate into most somatic cell lineages, and compatibility with precise genetic editing for the establishment of monoclonal cell lines that ensure phenotypic homogeneity [36, 37].

Thus, we developed an in vitro pyrogen detection model by engineering hESCs stably transfected with an NF‐κB‐regulated luciferase reporter system, wherein NF‐κB functions as the pyrogenic biomarker. Upon directed differentiation into hESC‐derived monocytes (hESC‐Mono), quantitative bioluminescent assays demonstrated high sensitivity in the detection of both endotoxins (e.g., LPS) and non‐endotoxin pyrogens (e.g., LTA and Zymosan). Collectively, this NF‐κB‐driven hESC‐Mono reporter platform represents a validated alternative to conventional pyrogen testing methodologies.

## 2 Materials and Methods

### 2.1 Construction of the NF‐κB Luciferase Plasmid (NF‐κB‐Luc)‐Puro

NF‐κB‐response element and luciferase sequences were amplified by PCR from the pSI‐Check2‐hRluc‐NFkB‐firefly plasmid (106979, Addgene). Subsequently, the construct was cloned into the AAVS1‐targeted vector using *Sal‐I* endonuclease.

### 2.2 Generation of a NF‐κB‐Reporter Clonal hESCs Line

Human embryonic stem cells (hESCs) were transfected using the Lonza P3 4D‐Nucleofector X Kit (V4XP‐3024, Lonza) according to the manufacturer's instructions. Briefly, hESCs were dissociated into single‐cell suspensions using TrypLE (A1285901, Thermo Fisher), resuspended in P3 Primary Cell Nucleofector Solution, and mixed with plasmids (AAVS1‐NF‐κB‐Luc‐puro and Cas9‐AAVS1‐sgRNA) in sterile centrifuge tubes. The cell‐plasmid mixture was electroporated using the 4D‐Nucleofector X System (Lonza) with program CA‐137. Immediately following electroporation, cells were gently resuspended in pre‐warmed E8 medium (A1517001, Gibco) and seeded onto a 6‐well plate coated with vitronectin (A14700, Thermo Fisher). Puromycin selection (150 ng/mL) was initiated 24 h post‐transfection and maintained for 48 h. Surviving cells were subsequently sorted by fluorescence‐activated cell sorting (Beckman) based on GFP expression driven by the AAVS1‐NF‐κB‐Luc‐puro construct. After 2–4 weeks of culture, genomic integration of NF‐κB‐Luc at the AAVS1 locus was confirmed by PCR amplification using primers listed in Table S1.

### 2.3 Cell Culture

The human monocytic leukaemia cell line THP‐1 (TIB‐202, ATCC) was maintained in suspension culture using RPMI 1640 medium (A1049101, Gibco) supplemented with 10% foetal bovine serum (FBS, 10091‐148, Gibco). Primary CD14^+^ human monocytes (purchased from Shanghai Yayu Bio.) were differentiated into macrophages by culturing in RPMI 1640 medium containing 10% FBS, 50 ng/mL recombinant human M‐CSF and 1% GlutaMAX (A12860‐01, Gibco) for 5 days. hESCs (CB0003), provided by the National Stem Cell Resource Center (NSCRC), Institute of Zoology, Chinese Academy of Sciences, were maintained in Essential 8 (E8) medium (A1517001, Gibco) on vitronectin‐coated 6‐well plates, with daily medium changes [38]. All cells were incubated at 37°C in a humidified atmosphere of 5% CO_2_. All cell lines were routinely tested for mycoplasma contamination.

### 2.4 Differentiation of Monocytes From hESCs

Monocyte differentiation from hESCs (hESC‐Mono) was performed according to a modified protocol based on the Sally A. Cowley team [39, 40]. Briefly, hESCs were plated at a density of 8000 cells per well in 96‐well ultra‐low attachment round‐bottom plates (7007, Corning) using E8 medium supplemented with 50 ng/mL BMP4, 50 ng/mL VEGF, 20 ng/mL SCF and 10 μM Y‐27632 (ROCK inhibitor). Plates were centrifuged at 150*g* for 5 min to promote aggregate formation. After 5 days, embryoid bodies (EBs) were collected and transferred to X‐Vivo 15 mediums (04‐418Q, Lonza) containing 2 mM GlutaMAX, 50 μM β‐mercaptoethanol (21985‐023, Gibco), 50 ng/mL M‐CSF and 25 ng/mL IL‐3. EBs were then seeded at a density of 1 EB/cm^2^ onto growth factor‐reduced Matrigel (12634‐010, Corning)‐coated plates (pre‐coated with Matrigel diluted in cold Advance DMEM/F12 at room temperature for 1 h). For the next 14 days, half‐medium changes were performed until CD14^+^ monocytes appeared in the supernatant. Subsequently, complete medium replacement was conducted every 3 days until terminal differentiation.

### 2.5 Macrophage Polarization

Monocytes were differentiated into macrophages by culturing in medium supplemented with 2 mM GlutaMAX, 1% penicillin–streptomycin (15140122, Gibco) and 50 ng/mL M‐CSF for 5 days. For M1 polarization, differentiated macrophages were stimulated with 50 ng/mL IFN‐γ (300‐02, PeproTech) and 50 ng/mL LPS (tlrl‐eklps, InvivoGen) in fresh X‐VIVO 15 media for 24 h. For M2 polarization, macrophages were treated with 50 ng/mL IL‐4 (200‐04, PeproTech) for 24 h. For M0 control, macrophages were maintained in medium with 50 ng/mL M‐CSF for 24 h.

THP‐1 cells were differentiated into macrophage‐like cells by treatment with 100 nM phorbol 12‐myristate 13‐acetate (PMA; P8139, Sigma‐Aldrich) in RPMI 1640 medium containing 10% foetal bovine serum and 1% penicillin–streptomycin for 24 h. Subsequent polarization was performed using the M1/M2/M0 protocols described above.

### 2.6 Flow Cytometry Analysis

Single‐cell suspensions of hESCs, THP‐1 cells, PBMC‐derived monocytes and hESC‐derived monocytes were prepared. Cell staining was performed as previously described [41]. Briefly, for surface marker staining, cells were incubated with fluorochrome‐conjugated antibodies targeting CD14, CD11B, CD31, CD34, CD43, CD44, CD80, CD86, CD206, CD45, HLA‐ABC and SSEA‐4 in FACS buffer (DPBS + 2% FBS) at 4°C for 15 min. For intranuclear staining of transcription factors NAONG and OCT3/4, cells were fixed and permeabilized using the Foxp3/Transcription Factor Staining Buffer Set (00‐5523‐00, Thermo Fisher), followed by incubation with intranuclear antibodies. Samples were analyzed on a CytoFLEX S flow cytometer (Beckman), and data were processed using FlowJo software (FlowJo LLC, BD Biosciences).

### 2.7 RNA Isolation, Reverse Transcription and Quantitative Real‐Time PCR

Total RNA was extracted from samples using the FastPure Cell/Tissue Total RNA Isolation Kit V2 (RC112‐01, Vazyme) according to the manufacturer's protocol. First‐strand cDNA was synthesized from total RNA using the PrimeScript 1st Strand cDNA Synthesis Kit (6110A, Takara Bio) with oligo dT primers. Quantitative real‐time PCR (qPCR) was performed on a LightCycler 480 II system (Roche) using the SYBR Green Real‐Time PCR Master Mix Plus (QPS‐201, Toyobo). Relative mRNA expression was calculated using the 2^−ΔΔCq^ method, normalized to *GAPDH* as the endogenous control. Primer sequences are listed in Table S1.

### 2.8 Luciferase Reporter Assay

The luciferase reporter assay was conducted using the Luciferase Reporter Assay System (E6120, Promega) according to the manufacturer's instructions. Briefly, hESCs or monocyte differentiation from hESCs were harvested, resuspended in culture medium and seeded onto 96‐well plates (3599, Corning). Pyrogen solutions diluted in culture medium (Essential 8 medium or X‐vivo15 medium) were added to designated wells. Plates were incubated at 37°C with 5% CO_2_ for the specified duration. Following incubation, substrate solution of One‐Glo Luciferase Assay System (Promega) was added (100 μL/well), followed by a 5‐min incubation at room temperature in the dark. Luminescence was subsequently measured using an EnSpire Multimode Plate Reader (PerkinElmer).

### 2.9 Cytokine Production Assays

Conditioned media were collected. Cytokine concentrations in the media were quantified using the Bio‐Plex Pro Human Screening Panel 9‐plex Assay (17010434, Bio‐Rad) according to the manufacturer's specifications. The analyzed cytokine profile included interleukin 1β (IL1β), IL6, IL8 and tumour necrosis factor α (TNFα). Concentrations that exceeded the detection limit were imputed as the highest value within the respective group.

### 2.10 Statistical Analysis

Statistical analyses were performed using GraphPad Prism 8.0 (GraphPad Software). All data are presented as the mean ± SD unless otherwise specified. For data with normal distribution and homogeneity of variance, an unpaired two‐tailed Student's *t*‐test, one‐way ANOVA, or two‐way ANOVA was applied as appropriate. For data that violated these assumptions, corrective analyses were employed, specifically non‐parametric tests (Mann–Whitney *U* test) or tests that do not assume equal variances (Welch's *t*‐test). The *p* value was calculated accordingly. NS, not significant; *p < 0.05; **p < 0.01; ***p < 0.001; ****p < 0.0001.

## 3 Results

### 3.1 hESC‐Derived Monocytes More Closely Resemble PBMC‐Derived Monocytes Compared to THP‐1 Cells

TLRs and their co‐factors have been reported to play a critical role in pyrogen‐induced fever and are highly expressed in monocytes [42]. However, primary monocytes derived from peripheral blood mononuclear cells (PBMC‐Mono) present challenges for genetic manipulation and limited scalability due to their restricted proliferative capacity. Moreover, the THP‐1 monocytic cell line exhibits aberrant expression of TLRs and co‐factors as a result of its malignant transformation. To overcome these limitations, we developed a serum‐free, chemically defined protocol to differentiate human pluripotent stem cells (hPSCs), including embryonic stem cells (hESCs) and induced pluripotent stem cells (hiPSCs), into monocytes, leveraging their unlimited self‐renewal and differentiation potential (Figure 1A) [39, 40]. The expression of TLRs and co‐factors (CD14 and MD2 encoded by *LY96*) was analyzed by RNA‐Seq (Table S2). The heatmap revealed that the TLR/co‐factor expression profile of hESC‐derived monocytes (hESC‐Mono) more closely resembled that of PBMC‐Mono compared to THP‐1 cells (Figure 1B). This finding was further validated by quantitative PCR (Figure 1C). Flow cytometry was performed to evaluate protein expression of monocyte markers CD14 and CD11B. In contrast to THP‐1 cells, hESC‐Mono cells exhibited a geometric mean fluorescence intensity (gMFI) of CD14 and CD11B that more closely resembled that of PBMC‐Mono (Figures 1D and S1A). Specifically, THP‐1 cells lacked detectable CD14 expression and showed markedly reduced CD11B surface levels (Figures 1D and S1A).

**FIGURE 1:**
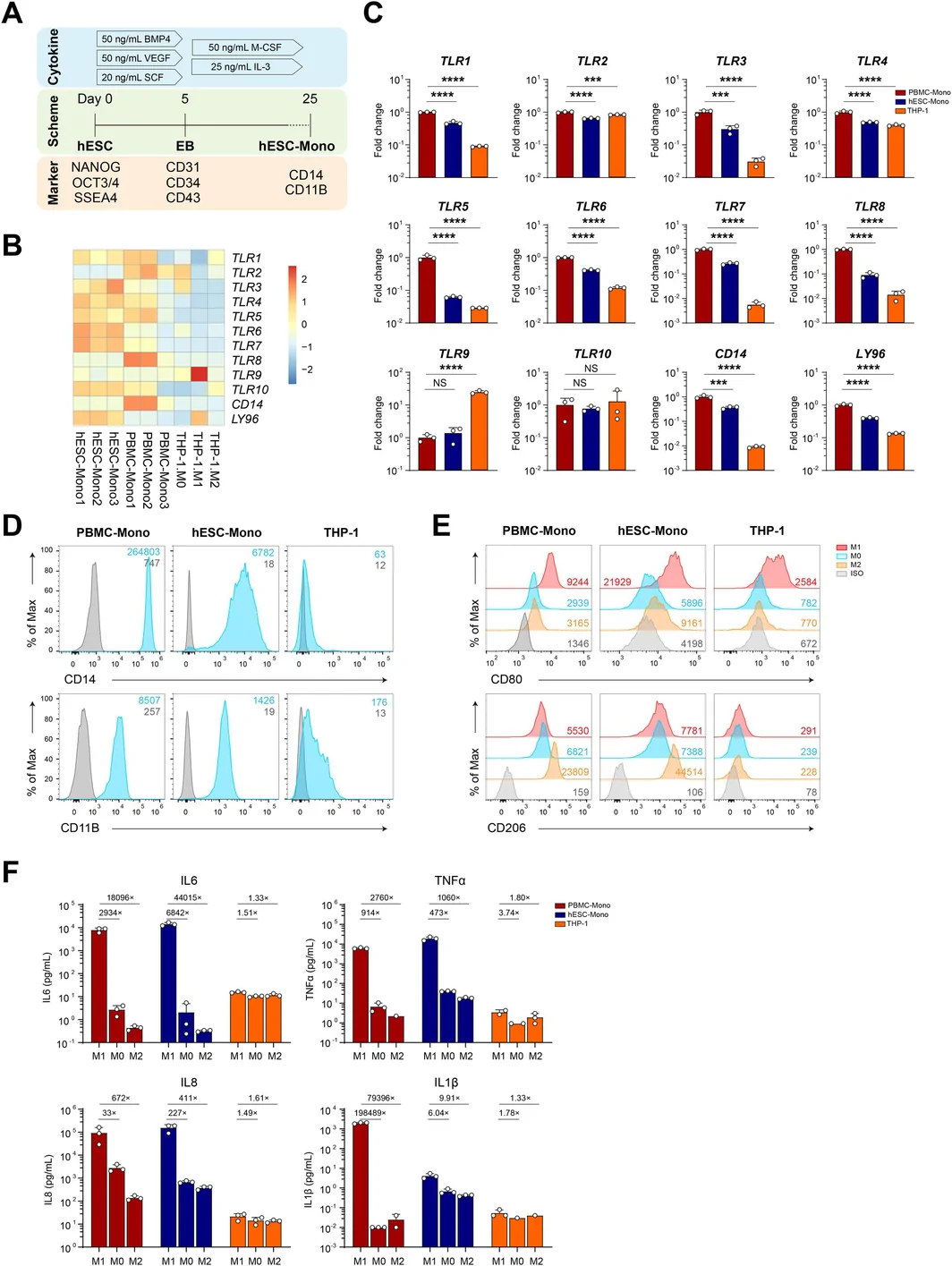
hESC‐derived monocytes exhibit comparable TLRs receptor expression profiles and functional responses to PBMC‐derived monocytes, distinct from THP‐1 cells. (A) Differentiation scheme of hESC‐derived monocytes (hESC‐Mono) and quality control markers at key stages. (B) Heatmap depicting the expression levels of Toll‐like receptors (TLRs) and co‐receptors in PBMC‐derived monocytes (PBMC‐Mono), hESC‐Mono and THP‐1 cells. (C) qPCR quantification of TLRs and co‐receptors expression in PBMC‐Mono, hESC‐Mono and THP‐1 cells. Data were normalized to PBMC‐Mono expression levels. Error bars represent SD (*n* = 3, mean ± SD). (D) Representative flow cytometry profiles of CD14 and CD11B surface markers in PBMC‐Mono (top), hESC‐Mono (middle) and THP‐1 cells (bottom). Grey histograms: Unstained controls; coloured curves: Antigen‐specific staining. (E) Polarization capacity assessment: CD80 (M1 marker) and CD206 (M2 marker) expression in PBMC‐Mono (left), hESC‐Mono (centre) and THP‐1 cells (right) following differentiation into M0 (unpolarized), M1 (LPS/IFNγ) and M2 (IL‐4) macrophage subsets. Representative flow cytometry was shown. (F) Pro‐inflammatory cytokine protein expression (IL6, TNFα, IL1β and IL8) in polarized macrophages. Three biological replicates were included per group. Samples below the detection limit were considered undetectable and were excluded from statistical analysis. Fold change values denote M1‐specific upregulation compared to both M0 and M2 subsets within each corresponding cell lineage, calculated as M1/M0 and M1/M2 ratios, respectively (mean ± SD). Error bars represent mean ± SD. ***p < 0.001; ****p < 0.0001; NS denotes not significant.

Polarization into M1 and M2 phenotypes is a critical functional capability of monocytes to respond to external stimuli and constitutes a key mechanism underlying pyrogen‐induced fever [43]. hESC‐Mono exhibited polarization profiles comparable to PBMC‐Mono, with LPS/IFN‐γ stimulation inducing high expression of CD80 (a canonical M1 marker) and IL‐4 treatment upregulating CD206 (a hallmark M2 marker) (Figures 1E and S1B). In contrast, THP‐1 cells demonstrated impaired polarization capacity, particularly for the M2 phenotype (Figures 1E and S1B). Cytokine secretion, a critical endpoint in the MAT for pyrogen detection, was further analyzed in polarized M1 and M2 cells. Upon M1 polarization, both PBMC‐Mono and hESC‐Mono secreted significantly higher levels of IL6, IL8 and TNFα compared to M0 or M2 macrophages (Figure 1F). However, M1‐polarized THP‐1 cells exhibited similarly high levels of IL8 expression compared to M0 and M2 macrophages but not IL6, TNFα, or IL1β. These results indicate that hESC‐Mono represents a more robust and physiologically relevant cellular platform for pyrogen detection assays compared to THP‐1 cells.

### 3.2 hESCs Engineered With the NF‐κB‐Luc Reporter System Retain Pluripotency and Maintain Haematopoietic Differentiation Capacity

To facilitate the detection of pyrogen response, we engineered a luciferase reporter gene driven by the NF‐κB response element (a key component of pyrogen‐induced fever signalling) and integrated it into the AAVS1 locus of hESCs via electroporation (Figure 2A). Genomic PCR spanning the homology arms (HA) confirmed precise integration at the AAVS1 locus (Figures 2B and S2). Furthermore, the NF‐κB‐Luc hESC line stably expressed GFP, thereby validating the robust integration of the reporter system (Figure 2C).

**FIGURE 2:**
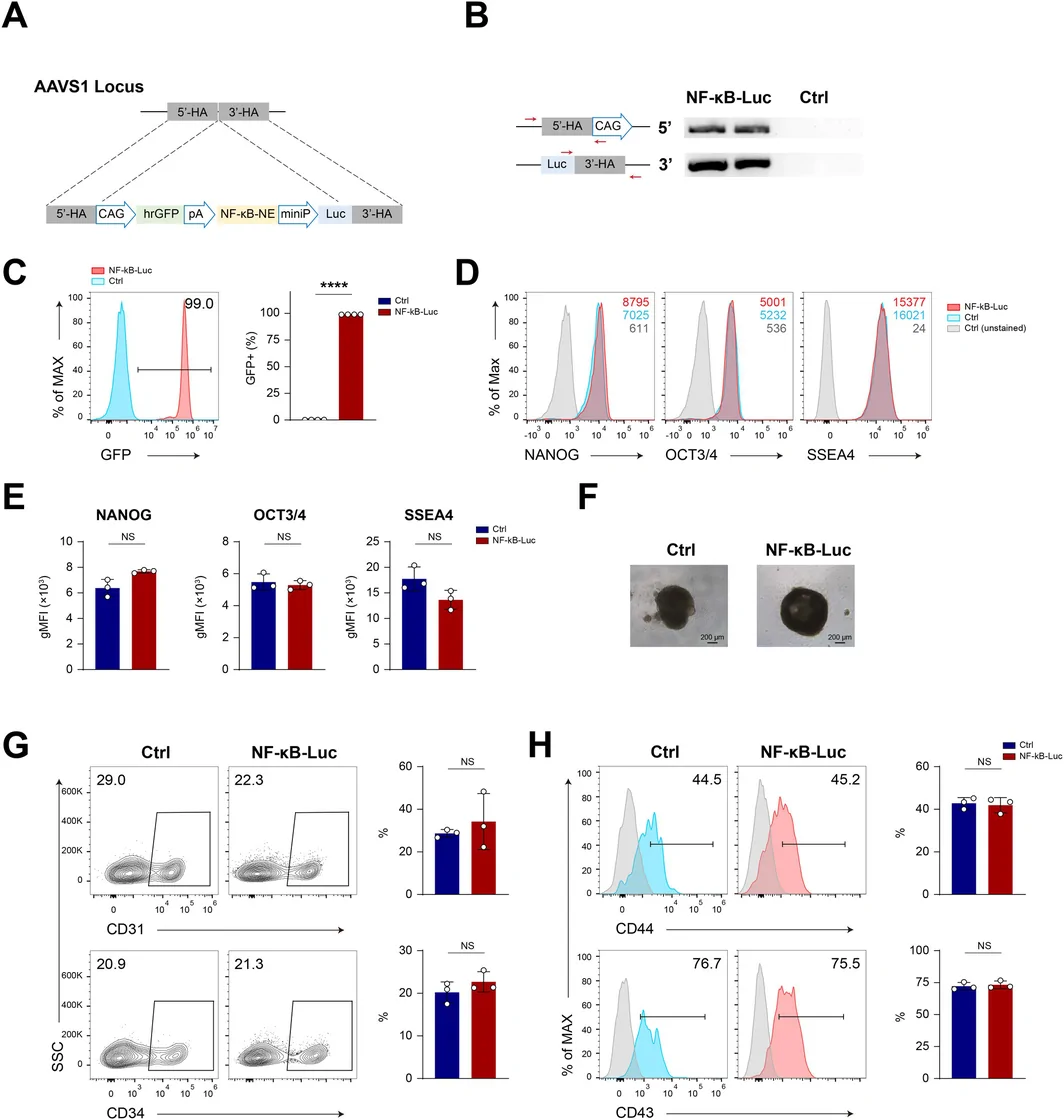
Phenotypic and functional characterization of NF‐κB reporter‐engineered hESC lines. (A) Schematic representation of the AAVS1 locus‐targeted integration strategy for the NF‐κB‐responsive luciferase reporter system (NF‐κB‐Luc). 5′HA/3′HA: 5′/3′ homology arms. (B) Genomic validation of NF‐κB‐Luc integration by PCR agarose gel electrophoresis confirms precise targeting at 5′ and 3′ homology regions in hESC clones (Ctrl: Parental hESCs as control). (C) Flow cytometric quantification of GFP^+^ hESCs following puromycin selection demonstrates stable integration of the reporter cassette. Filled histograms depict un‐transfected control (blue) versus NF‐κB‐Luc hESCs (red). The percentages of GFP^+^ cells are shown in the right panel (*n* = 4, mean ± SD). (D, E) Pluripotency marker profiling. Intranuclear staining for NANOG (left) and OCT3/4 (centre) and surface staining for SSEA4 (right) in NF‐κB‐Luc hESCs or Ctrl (D). Grey histograms: Unstained controls. The geometric mean fluorescence intensity (gMFI) of NANOG, OCT3/4 and SSEA4 is shown in panel (E) (*n* = 3, mean ± SD). (F) Phase‐contrast microscopy images of day‐5 embryoid bodies (EBs) derived from Ctrl hESCs (left) or NF‐κB‐Luc hESCs (right). Scale bars: 200 μm. (G, H) Assessment of haematopoietic differentiation capacity. Flow cytometry plots show CD31^+^/CD34^+^ endothelial‐haematopoietic progenitors (G) and CD44^+^/CD43^+^ myeloid progenitors (H) in day‐5 EBs. The percentages of each cell subset are shown in the right panel (*n* = 3, mean ± SD). Data are representative of at least three independent experiments. Error bars represent mean ± SD. ****p < 0.0001; NS denotes not significant.

To evaluate the potential effects of reporter insertion on hESC properties, we analyzed the expression of pluripotency markers. NF‐κB‐Luc hESCs maintained expression levels of key pluripotency markers (NANOG, OCT3/4 and SSEA‐4) comparable to those of parental hESCs (used as controls) (Figure 2D,E). Monocyte differentiation from hESCs proceeds through sequential developmental stages, including mesoderm specification, haematopoietic commitment and myeloid progenitor formation [39]. To assess the differentiation capacity of NF‐κB‐Luc hESCs, we generated embryoid bodies (EBs) and evaluated their formation efficiency. Results demonstrated that NF‐κB‐Luc hESCs successfully differentiated into EBs with morphology and size comparable to wild‐type controls (Figure 2F). Surface marker expression was monitored via flow cytometry to track progression through critical stages: endothelial progenitors (CD31^+^), haematopoietic progenitors (CD34^+^) and myeloid progenitors (CD43^+^/CD44^+^). We found that NF‐κB‐Luc hESCs exhibited normal differentiation kinetics comparable to controls, with no significant differences in marker expression profiles (Figure 2G,H). These results confirmed that hESCs with targeted integration of the reporter system retain the capacity to differentiate into haematopoietic lineages.

### 3.3 Genetically Modified NF‐κB‐Luc hESC‐Mono Retain Polarization Capability and Cytokine Secretion Capacity

To evaluate the differentiation potential of NF‐κB‐Luc‐engineered hESCs into functional monocytes, we performed directed differentiation to generate hESC‐Mono and characterized their phenotypic and functional properties. Flow cytometry analysis revealed that CD11B^+^CD14^+^ monocytes constituted approximately 98% of the total suspension cell population (Figure 3A). The differentiated hESC‐Mono stably expressed GFP, confirming transgene retention, in contrast to unmodified control cells (Figure 3B). Furthermore, hESC‐Mono exhibited canonical monocyte surface markers, including CD206, CD45, CD43, CD86 and HLA‐ABC (Figure 3C,D). TLRs and co‐receptor expression profiles were largely unaffected by NF‐κB‐Luc integration, with the exception of *TLR5* and MD2 (*LY96*) (Figure 3E). Functional assays demonstrated that hESC‐Mono retained robust responsiveness to LPS/IFN‐γ stimulation, as evidenced by polarization into M1 macrophages (Figure 3F) and secretion of pro‐inflammatory cytokines (Figure 3G). These findings indicate that NF‐κB‐Luc modification does not compromise the differentiation potential, phenotypic identity, or functional capacity of hESC‐Mono.

**FIGURE 3:**
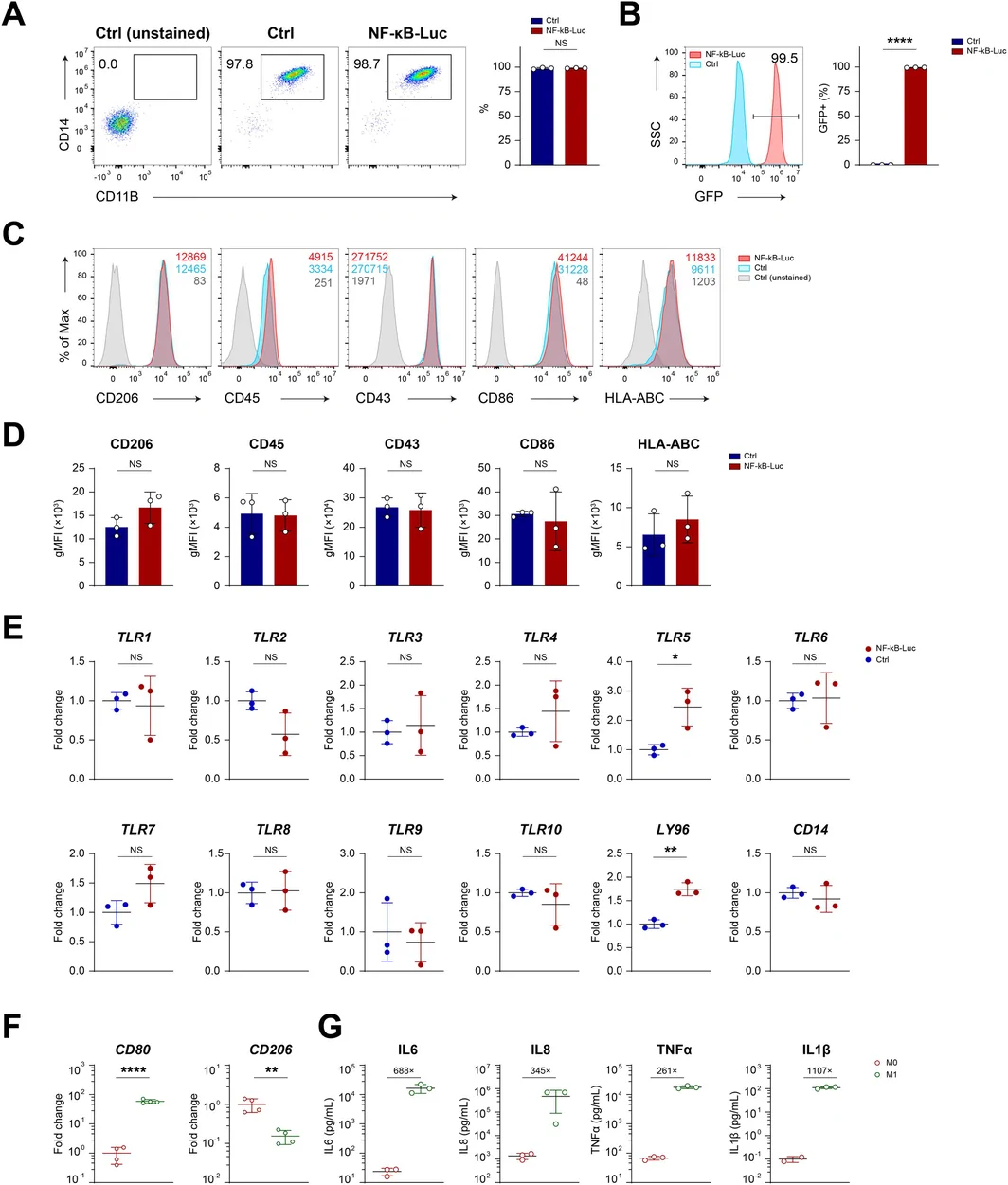
Assessment of the differentiation and response capabilities of monocytes derived from NF‐κB‐Luc‐engineered hESCs. (A) Validation of monocyte differentiation purity. Flow cytometric quantification of CD14^+^CD11B^+^ populations in NF‐κB‐Luc hESC‐Mono (right) compared to un‐transfected controls (centre). The unstained control is shown on the left side. The percentages of CD14^+^CD11B^+^ double positive cells are shown in the right panel (*n* = 3). (B) Evaluation of reporter stability. Representative flow cytometry histograms showing constitutive GFP expression in NF‐κB‐Luc hESC‐Mono (red) versus GFP‐negative un‐transfected controls (blue). The percentages of GFP^+^ cells are shown in the right panel (*n* = 3). (C, D) Profiling of monocytic surface marker. Representative histograms (C) show the expression of CD206, CD45, CD43, CD86 and HLA‐ABC expression in NF‐κB‐Luc (red) and Ctrl hESC‐Mono (blue) by flow cytometry. Grey histograms: Unstained controls. The gMFI of CD206, CD45, CD43, CD86 and HLA‐ABC is shown in panel (D). (E) Competence of TLR signalling. qPCR analysis of TLR1‐10 and co‐receptor (CD14/MD2) mRNA levels in NF‐κB‐Luc hESC‐Mono (red) versus Control (blue). Data were normalized to un‐transfected controls (*n* = 3, mean ± SD). (F) qPCR analysis of M1 (CD80) and M2 (CD206) polarization markers in LPS/IFN‐γ‐stimulated macrophages. Data were normalized to M0 controls (*n* = 4, mean ± SD). (G) Pro‐inflammatory cytokine protein levels (IL6, TNFα, IL1β and IL8) post‐polarization. Three biological replicates were included per group. Samples below the detection limit were considered undetectable and were excluded from statistical analysis. Fold‐changes were calculated relative to M0 macrophages (mean ± SD). Data are representative of at least three independent experiments. Error bars represent mean ± SD. *p < 0.05; **p < 0.0; ****p < 0.0001; NS denotes not significant.

### 3.4 The NF‐κB‐Luc hESC‐Mono System Is Capable of Reporting at Least Three Classes of Pyrogens

In pyrogen detection assays, achieving a high signal‐to‐noise ratio (SNR) is critical for ensuring low limits of detection (LOD), particularly in the context of trace endotoxin quantification. As shown in Figure 4A, luciferase activity was specifically detected in NF‐κB‐Luc‐engineered hESC‐Mono but not in untransfected hESC‐Mono controls or hESCs (including both wild‐type and NF‐κB‐Luc hESCs). The NF‐κB‐Luc hESC‐Mono system demonstrated an exceptionally high SNR for LPS, thereby enabling reliable discrimination between true signals and background noise. These results confirm the potential of stably transfected NF‐κB‐Luc hESC‐Mono cells for sensitive pyrogen detection.

**FIGURE 4:**
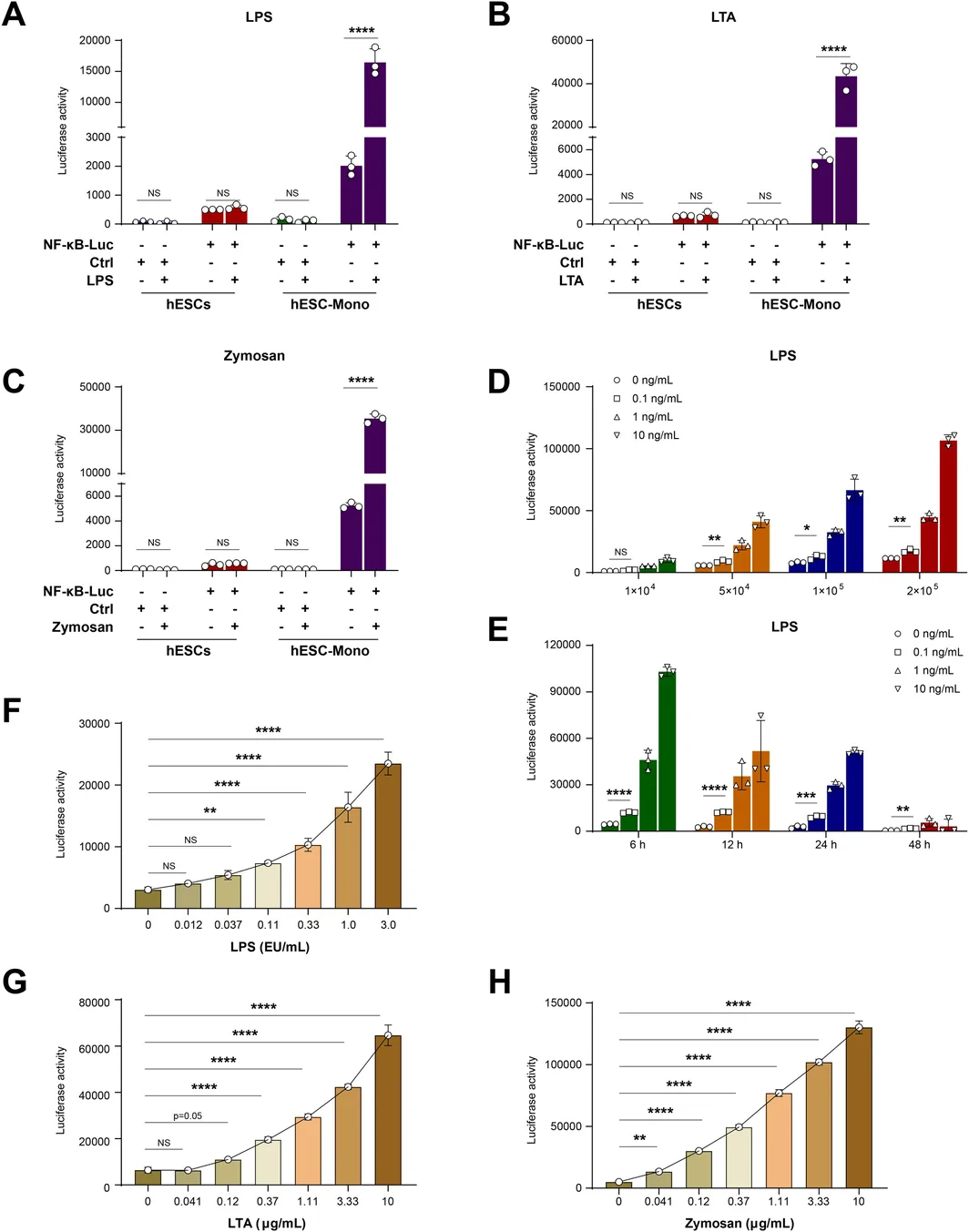
NF‐κB‐Luc‐engineered hESC‐Mono exhibit dose‐ and time‐dependent responsiveness to three classes of pyrogens. (A–C) Pyrogen‐induced NF‐κB activation. Luciferase activity in hESCs (Ctrl or NF‐κB‐Luc) and hESC‐Mono (Ctrl or NF‐κB‐Luc), following 6‐h stimulation with 100 ng/mL LPS (A), 10 μg/mL LTA (B), or 10 μg/mL Zymosan (C) (*n* = 3, mean ± SD). (D) Kinetics of NF‐κB activation across varying cell densities (1 × 10^4^, 5 × 10^4^, 1 × 10^5^, 2 × 10^5^ cells/well) following LPS exposure (0–10 ng/mL, 6 h). Bar graphs represent luciferase activity (*n* = 3, mean ± SD). (E) Time‐course profiling. Concentration‐dependent NF‐κB activation by LPS (0–10 ng/mL) in 1 × 10^5^ cells/well across 6–48 h of post‐stimulation. Bar graphs represent luciferase activity (*n* = 3, mean ± SD). (F) Dose–response analysis. Luciferase activity in response to Chinese Pharmacopoeia‐certified LPS standards (0.012–3.0 EU/mL) at 1 × 10^5^ cells/well after 6 h of incubation (*n* = 3, mean ± SD). (G) Dose–response analysis. Dose–response to LTA (0.041–10 μg/mL) at 1 × 10^5^ cells/well after 6 h of incubation (*n* = 3, mean ± SD). (H) Dose–response analysis. Dose–response curves to Zymosan (0.041–10 μg/mL) at 1 × 10^5^ cells/well after 6 h of incubation (*n* = 3, mean ± SD). Data are representative of at least three independent experiments. Error bars represent mean ± SD. *p < 0.05; **p < 0.01; ***p < 0.001; ****p < 0.0001; NS denotes not significant.

To further investigate whether the system is capable of detecting both Gram‐negative bacterial endotoxins and Gram‐positive bacterial non‐endotoxins, we evaluated the response to lipoteichoic acid (LTA), a key structural component of Gram‐positive bacterial cell walls. Results demonstrated that NF‐κB‐Luc hESC‐Mono exhibited an exceptionally high signal‐to‐noise ratio (SNR) for LTA compared to control hESC‐Mono cells or hESCs (Figure 4B), demonstrating the system's potential to detect non‐endotoxin LTA. To broaden the detection spectrum of the system, we evaluated its responsiveness to Zymosan, a fungal pyrogen derived from yeast cell walls. Compared to hESCs and control hESC‐Mono cells, NF‐κB‐Luc hESC‐Mono exhibited robust reporter activity in response to Zymosan (Figure 4C). Collectively, these findings indicate that NF‐κB‐Luc hESC‐Mono reliably detects three classes of pyrogens: Gram‐negative endotoxin (LPS), Gram‐positive cell wall component (LTA) and fungal Zymosan.

Furthermore, we previously observed that hESC‐mono cells express TLR7 and TLR8, which are primarily localized to the endosomal membrane and recognize single‐stranded RNA, playing a critical role in the detection of RNA viruses. Given this, we sought to determine whether these cells possess a functional TLR7/8 signalling axis capable of activating our reporter system. To this end, we challenged the cells with TLR7/8 agonists. As expected, both Resiquimod (R848), a dual TLR7/8 agonist and Imiquimod (R837), a selective TLR7 agonist, robustly activated the reporter (Figure S3A,B). These results indicate that the NF‐κB‐Luc hESC‐Mono is responsive to TLR7/8 activation, suggesting its potential utility in detecting viral‐associated molecular patterns.

### 3.5 The NF‐κB Response Element‐Mediated Reporter System Exhibits High Detection Sensitivity

Cell seeding density and incubation time are critical factors that determine LOD of the reporter system. To optimize these parameters, we systematically evaluated their effects on the NF‐κB response in hESC‐Mono. Increasing cell density elevated background signals in negative controls. However, at low LPS concentrations (0.1 ng/mL), all tested densities (5 × 10^4^ to 2 × 10^5^ cells/well) exhibited significantly higher NF‐κB responses compared to the negative control (*p* < 0.05) (Figure 4D). Extending stimulation duration revealed that absolute NF‐κB responses peaked at 6–24 h across LPS concentrations, after which response signals progressively decreased over time (Figure 4E). The relative NF‐κB response (normalized to the negative control) to high‐concentration LPS (10 ng/mL) reached its maximum at 6 h. Based on these findings, optimal assay conditions were defined as 1 × 10^5^ cells/well and 6 h of stimulation. We then evaluated the detection limit using an LPS reference standard obtained from the China National Institutes for Food and Drug Control (NIFDC; code: 150800), with each vial containing 9000 EU (endotoxin unit). The results demonstrated that LPS induced a stable dose‐dependent activation of NF‐κB in hESC‐Mono. The detection limit was determined to be 0.037 EU/mL according to the criterion specified in the European Pharmacopoeia [Average (+LPS) > Average (blank) + 3 × SD (blank)], which is a standard method for assessing assay sensitivity. It should be noted that, although the response at this concentration met the pharmacopoeia criterion for detection, it did not achieve statistical significance (*p* ≥ 0.05) when compared to the blank control by one‐way ANOVA (Figure 4F).

We also assessed the impact of cell density on non‐endotoxin detection. Four cell suspensions (1 × 10^4^, 5 × 10^4^, 1 × 10^5^ and 2 × 10^5^ cells/well) were prepared and stimulated with non‐endotoxin LTA or Zymosan at concentrations ranging from 0 to 10 μg/mL. Following a 6‐h incubation period, luciferase activity was measured. Significant reporter activity was detected at 0.1 μg/mL for both LTA and Zymosan across all tested cell densities (Figure S3C,E). However, considering the fold differences and luciferase activity values, we selected a cell density of 1 × 10^5^ cells/mL for subsequent non‐endotoxin time‐ and dose‐dependent assays. Regarding detection time, similar to LPS stimulation, detectable NF‐κB responses to LTA or Zymosan in hESC‐Mono peaked between 6 and 24 h, with peak activity observed at 6 h (Figure S3D,F). The limit of detection for LTA was determined to be 0.12 μg/mL (*p* < 0.05 vs. negative control) (Figure 4G). The system exhibited high sensitivity to Zymosan, maintaining reporter activity at concentrations as low as 0.04 μg/mL (Figure 4H).

### 3.6 Distinct Classes of Pyrogens Elicit Specific Immune Response Profiles

Although NF‐κB‐Luc hESC‐Mono exhibit high sensitivity to non‐endotoxins, their response is significantly lower than that to LPS. To further investigate the mechanisms underlying this phenomenon, we analyzed the expression levels of activation‐related markers and pro‐inflammatory factors. Upon LPS stimulation, hESC‐Mono not only upregulated the expression of the co‐stimulatory molecule CD80 but also downregulated the expression of the marker CD206 (Figure 5A). These results confirm that NF‐κB‐Luc‐engineered hESC‐Mono cells retain robust responsiveness to LPS. In contrast to LPS, LTA stimulation did not significantly upregulate the co‐stimulatory molecule CD80 but reduced the expression of the mannose receptor CD206, confirming specific activation of the NF‐κB‐Luc hESC‐Mono system by LTA (Figure 5B). Similarly, Zymosan stimulation also reduced CD206 expression without altering CD80 levels (Figure 5C).

**FIGURE 5:**
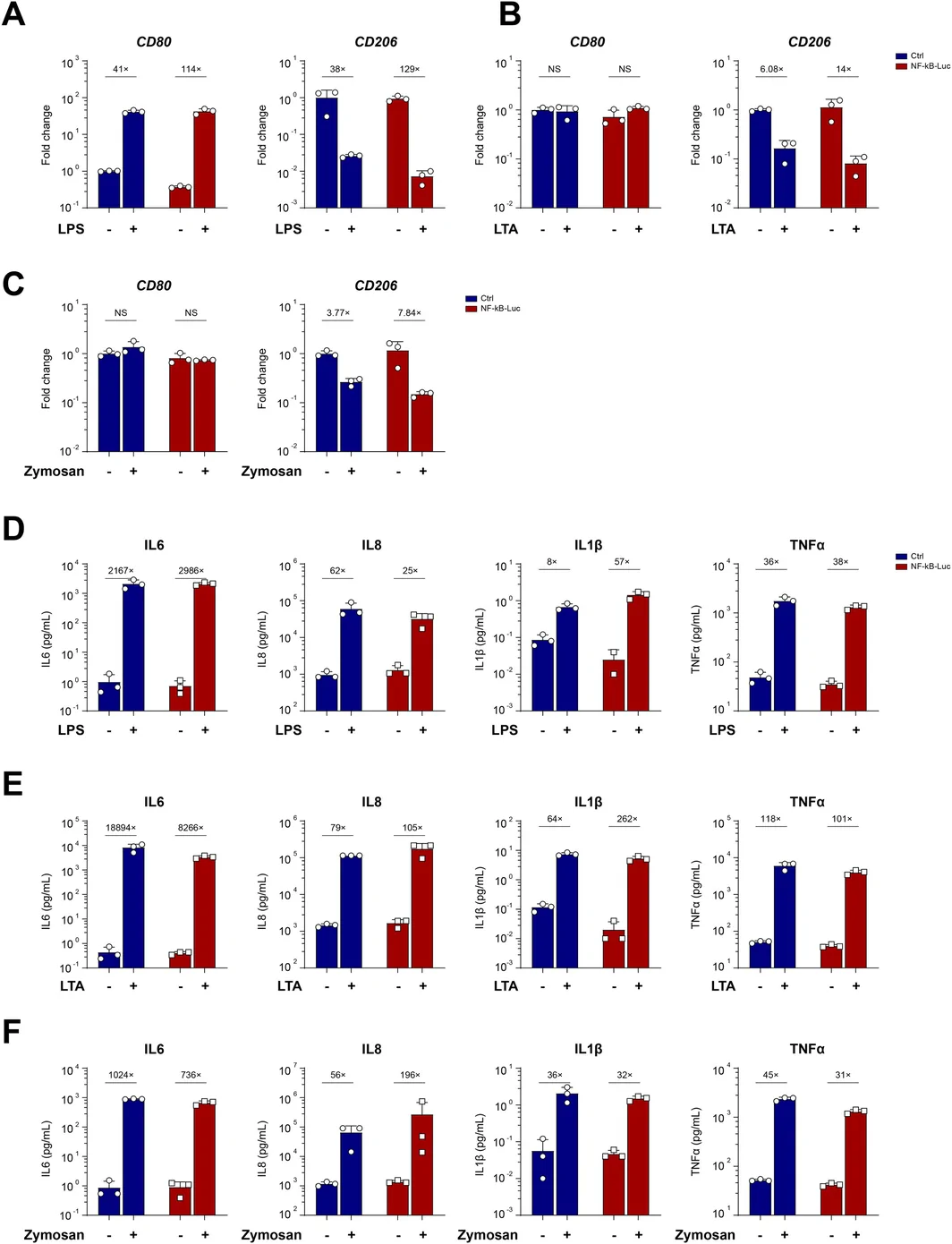
Different pyrogens induce distinct gene and cytokine expression profiles. (A–C) qPCR quantification of CD80 (M1) and CD206 (M2) markers in NF‐κB‐Luc hESC‐Mono cells (red) or un‐transfected hESC‐Mono cells (ctrl, blue) stimulated with 10 ng/mL LPS (A), 10 μg/mL LTA (B), or 10 μg/mL Zymosan (C). Data were normalized to unstimulated hESC‐Mono controls. Fold‐changes were calculated relative to unstimulated hESC‐Mono (*n* = 3, mean ± SD). (D) LPS‐induced pro‐inflammatory cytokine secretion. Protein levels of IL6, TNFα, IL1β and IL8 in hESC‐Mono following stimulation with LPS. Three biological replicates were included per group. Samples below the detection limit were considered undetectable and were excluded from statistical analysis. Fold‐changes were calculated relative to unstimulated hESC‐Mono (mean ± SD). (E) LTA‐induced pro‐inflammatory cytokine secretion. Protein levels of IL6, TNFα, IL1β and IL8 in hESC‐Mono following stimulation with LTA. Fold‐changes were calculated relative to unstimulated hESC‐Mono (*n* = 3, mean ± SD). (F) Zymosan‐induced pro‐inflammatory cytokine secretion. Protein levels of IL6, TNFα, IL1β and IL8 in hESC‐Mono following stimulation with Zymosan. Fold‐changes were calculated relative to unstimulated hESC‐Mono (*n* = 3, mean ± SD). Error bars represent mean ± SD.

Meanwhile, the expression levels of cytokines induced by endotoxins and non‐endotoxins also exhibit specificity. Although both LPS and the non‐endotoxin stimuli LTA and Zymosan induced high‐level secretion of the pro‐inflammatory cytokines IL6, IL8, IL1β and TNFα (Figure 5D–F), stimulation with 10 μg/mL LTA led to higher levels of pro‐inflammatory cytokine secretion compared to 10 μg/mL Zymosan, particularly for IL6, with the exception of IL8. Stimulation with 10 μg/mL Zymosan resulted in secretion levels of IL8, IL1β and TNFα comparable to those induced by 10 ng/mL LPS; however, LPS stimulated higher levels of IL6 secretion. These findings demonstrate stimulus‐specific regulation of pathways, surface markers and cytokine profiles by different pyrogens, thereby contributing to differential detection sensitivity of hESC‐Mono to various pyrogens.

## 4 Discussion

Peripheral blood monocytes (PBMC‐Mono) have long been regarded as the optimal choice for pyrogen detection due to their physiological relevance to human fever mechanisms and pathogen recognition profiles [2, 8]. However, their non‐proliferative nature and inherent challenges in genetic manipulation necessitate the development of alternative monocyte‐like cell systems [44]. Human pluripotent stem cell‐derived monocytes (hPSC‐Mono) have emerged as a promising candidate, with recent methodological advancements significantly improving differentiation efficiency, cost‐effectiveness and functional maturity [39, 45, 46, 47, 48]. Comparative analyses have demonstrated that hPSC‐Mono exhibit surface marker profiles comparable to those of PBMC‐derived monocytes, along with similar phagocytic capacity and polarization dynamics (M1/M2). Notably, hPSC‐Mono retains therapeutic potential through chimeric antigen receptor (CAR) engineering, mirroring the applications of PBMC‐Mono in tumour targeting and fibrotic disease intervention [49, 50, 51]. Recent breakthroughs in scalable bioreactor‐based production systems further enable standardized manufacturing, effectively addressing issues related to batch‐to‐batch variability, donor heterogeneity and ethical constraints associated with primary cell sources [52, 53, 54].

While the Lachmann group analyzed IL‐6 secretion levels in iPSC‐derived macrophages (following terminal differentiation of monocytes for 6 days) upon pyrogen stimulation [55], whether their precursor monocytes possess pyrogen detection capabilities, TLR expression profiles and responsiveness to various pyrogens following reporter system engineering remain unvalidated. Therefore, our study establishes the hESC‐based pyrogen detection model, providing an alternative approach and a solid theoretical foundation for advancing industrial applications of in vitro pyrogen detection methods.

The human immune system employs 10 TLRs that exhibit compartmentalized pathogen recognition strategies [56]. Cell surface TLRs (TLR1, TLR2, TLR4, TLR5 and TLR6) specialize in detecting lipid and protein components, while endosomal TLRs (TLR3, TLR7, TLR8, TLR9 and TLR10) mediate nucleic acid sensing. Specifically, the TLR4‐CD14‐MD2 complex orchestrates endotoxin (LPS) recognition through coordinated molecular interactions [57, 58, 59]. Non‐endotoxic pyrogens lipoteichoic acid (LTA) and Zymosan are detected via distinct TLR2 heterodimers: TLR2/1 and TLR2/6 [60, 61, 62, 63]. Besides, Zymosan also reacts via Dectin‐1. Beyond the canonical TLRs responsive to these three pyrogens, hESC‐Mono also express an expanded TLR repertoire, including TLR5 (bacterial flagellin detection), TLR3 (viral double‐stranded RNA sensing), TLR7/8 (microbial/viral ssRNA recognition) and TLR9 (CpG DNA responsiveness) [34]. Crucially, all TLR signalling cascades converge on NF‐κB pathway activation—the universal transcriptional hub for inflammatory responses. This conserved signalling architecture predicts that our NF‐κB‐Luc hESC‐Mono system will exhibit pan‐TLR responsiveness, generating dose‐dependent luminescent signals proportional to pyrogen concentrations across bacterial (LPS/LTA/flagellin), fungal (Zymosan) and viral (dsRNA/ssRNA) components.

The NF‐κB response element‐driven luciferase reporter system enables quantitative measurement of NF‐κB pathway activation, facilitating the development of a rapid pyrogen detection platform [1, 64]. The conventional PBMC‐based MAT requires co‐incubation with pyrogens for 24–48 h, followed by ELISA to measure pro‐inflammatory cytokine secretion (ChP 2020, General Rule 9301). ELISA is a time‐consuming and labour‐intensive procedure. In contrast, the NF‐κB‐Luc hESC‐Mono system requires only 6 h of co‐incubation with pyrogens, after which luminescence can be measured within minutes following substrate addition. This approach not only greatly shortens the assay time but also eliminates the extensive manual handling associated with ELISA.

While all pyrogens activate NF‐κB through TLRs, the signalling intensity and pathway diversity vary significantly among receptor subtypes. Upon LPS stimulation, TLR4 employs dual activation mechanisms: the MyD88‐dependent pathway initiates early‐phase NF‐κB responses, whereas the MyD88‐independent pathway sustains late‐phase activation [65, 66]. In contrast, TLR7 and TLR9 exhibit strict MyD88 dependence for NF‐κB signalling [33, 67]. This mechanistic divergence underpins the differential NF‐κB‐Luc reporter sensitivity and cytokine profiles observed in our study (and corroborated by prior reports [1, 32, 64]) across distinct pyrogen classes, particularly between endotoxin (LPS) and non‐endotoxin (LTA and Zymosan) activators.

Furthermore, current pyrogen testing predominantly relies on purified endotoxin (LPS) and non‐endotoxin pyrogen reagents (LTA, Zymosan), which differ significantly from naturally occurring pathogens that exist as intact bacteria, viruses, or microbial complexes. These whole microorganisms present polyvalent pathogen‐associated molecular patterns (PAMPs) extending beyond cell wall components such as LPS; their genomic nucleic acids also activate complementary TLRs [33]. In contrast, our endotoxin preparations represent single molecular species and may therefore lack the physiological complexity of natural mixtures documented in pyrogen testing for parenteral pharmaceuticals and medical devices. To bridge this translational gap, future efforts will require rigorous validation of the platform's sensitivity and accuracy in real‐world application scenarios, benchmarked against conventional pyrogen detection methods, such as LAL and RPT assays.

Therefore, this study established a novel pyrogen detection platform based on hESC‐Mono. Through directed differentiation of hESCs, we generated functional monocytes demonstrating phenotypic and functional equivalence to PBMC‐Mono, while avoiding the neoplastic transformation artefacts inherent in immortalized cell lines. Site‐specific integration of an NF‐κB‐responsive luciferase reporter at the AAVS1 safe harbour locus preserved pluripotency markers in engineered hESCs and maintained their capacity for monocytic differentiation. The NF‐κB‐Luc hESC‐Mono system demonstrated pyrogen reporting capabilities, exhibiting dose‐dependent responses to LPS, LTA and Zymosan. Notably, the platform achieved an LPS detection limit significantly below both the 0.5 EU/mL safety threshold for intravenous infusion and the 0.2 EU/mL threshold for intrathecal administration mandated by the Chinese Pharmacopoeia (ChP 2020, General Rule 1143), as validated via the LAL test and RPT assay. Future monoclonal subcloning may yield clones with higher LPS sensitivity. While industrial application requires further validation, our study provides a novel conceptual framework and cellular platform for pyrogen detection—a hPSC‐based system designed to achieve batch‐to‐batch consistency, enhanced sensitivity and ethical compliance through animal‐independent methodology. This innovation addresses critical limitations of conventional animal‐dependent assays (RPT and LAL) by eliminating inter‐species variability while maintaining human TLR signalling fidelity.

## 5 Conclusion

In summary, we have developed a monocyte‐based reporter system for interrogating the NF‐κB signalling axis—a central pathway governing pyrogenic responses. This platform demonstrates rapid, dose‐dependent detection of both endotoxins (LPS) and non‐endotoxins (LTA and Zymosan), with quantification robustness experimentally validated across relevant concentrations. By overcoming the operational complexity, protracted timelines and cell number limitations inherent to conventional MAT, this system provides a mechanistically grounded alternative for pyrogen screening.

## Author Contributions

J.W., W.L., Q.Z. and J.L. designed and supervised the experiments. J.L., B.C., T.G. and W.W. performed the experiments and analyzed the data throughout the study. L.W., J.H. and Q.Z. provided conceptual guidance. J.L. and J.W. wrote the manuscript with input from all authors.

## Funding

This work was supported by the Strategic Priority Research Program of the Chinese Academy of Sciences (XDC0200000, XDA0510000), National Key Research and Development Program of China (2023YFC3605100, 2020YFA0804000, 2021YFA1101600), National Natural Science Foundation of China (32225030, 82488301, 32370851, 32400604), and Beijing Natural Science Foundation (Z240018).

## Ethics Statement

The hESCs (CB0003) were purchased from National Stem Cell Resource Center (NSCRC), Institute of Zoology, Chinese Academy of Sciences (Beijing, China). The original source (NSCRC, http://cascrm.ioz.ac.cn/hotResourceCenterCell) has confirmed that there was initial ethics approval for collection of hESCs and that the donors had signed informed consent.

## Conflicts of Interest

The authors declare no conflicts of interest.

## Supporting information


**Figure S1:** hESC‐derived monocytes exhibit comparable TLRs receptor expression profiles and functional responses to PBMC‐derived monocytes, distinct from THP‐1 cells.
**Figure S2:** Confirmation of NF‐κB‐Luc reporter integration site.
**Figure S3:** Determination of optimal assay conditions for NF‐κB‐Luc hESC‐Mono response to non‐endotoxin stimuli.

**Table S1:** The primer list of PCR and qPCR.

**Table S2:** RNA‐seq raw read count matrix for hESC‐Mono, PBMC‐Mono and THP‐1.

## Data Availability

The data that support the findings of this study are available in the Supporting Information of this article.
