# Supplementary material for: Monocytes Derived From Human Pluripotent Stem Cells Engineered for Detection of Pyrogens
Source: Cell Prolif. 2026 May 21;59(10):e70233. doi: 10.1111/cpr.70233 (PMC13325647; doi:10.1111/cpr.70233)
Supplement: Supplementary file 1 [file CPR-59-e70233-s001.docx]

Supplementary Table 1

Table S1. The primer list of PCR and qPCR

| **Name** | **Sequence 5’ → 3’** |
| --- | --- |
| **Primers for PCR** | |
| 5’-HA-F | TAACGCTGCCGTCTCTCTCCTGAG |
| 5’-HA-R | ACCGTGGGCTTGTACTCGGT |
| 3’-HA-F | GGGTGCCTAATGAGTGAGCTAACTC |
| 3’-HA-R | AAAGGCAGCCTGGTAGACAGGGCTG |
| **Primers for qPCR** | |
| *CD80*-F | GGGAACATCACCATCCAAGT |
| *CD80*-R | CATTGTGACCACAGGACAGC |
| *CD206*-F | TCCGGGTGCTGTTCTCCTA |
| *CD206*-R | CCAGTCTGTTTTTGATGGCACT |
| *TLR1*-F | CCACGTTCCTAAAGACCTATCCC |
| *TLR1*-R | CCAAGTGCTTGAGGTTCACAG |
| *TLR2*-F | ATCCTCCAATCAGGCTTCTCT |
| *TLR2*-R | GGACAGGTCAAGGCTTTTTACA |
| *TLR3*-F | CGATTCCTTTGCTTGGCTTCC |
| *TLR3*-R | GGGAGTGAGGCAAGGGAAAT |
| *TLR4*-F | AGACCTGTCCCTGAACCCTAT |
| *TLR4*-R | CGATGGACTTCTAAACCAGCCA |
| *TLR5*-F | CGCTTCTCCTCCTGTAGTGG |
| *TLR5*-R | CCCTTAATGCAGTCAGATGGC |
| *TLR6*-F | TTCAGTTTCCCACCCATCGG |
| *TLR6*-R | GCCAGCCCTCTAACACTTCA |
| *TLR7*-F | CGAACCTCACCCTCACCATT |
| *TLR7*-R | GCCTCTTGATGCACATGTTGT |
| *TLR8*-F | ATGTTCCTTCAGTCGTCAATGC |
| *TLR8*-R | TTGCTGCACTCTGCAATAACT |
| *TLR9*-F | CGCTGCCCAAATCCCTCATA |
| *TLR9*-R | GTGACAGGTGGGTGAGGTTG |
| *TLR10*-F | CAGAGGGTGATGCTCCAGAG |
| *TLR10*-R | AATCCAGTGTCGTTGTGGCT |
| *MD2*-F | GGGTCTGCAACTCATCCGAT |
| *MD2*-R | CGTCATCAGATCCTCGGCAA |
| *CD14*-F | AGCCTTCCAGTGTGTGTCTG |
| *CD14*-R | TCGAGCGTCAGTTCCTTGAG |
