# Supplementary material for: Monocytes Derived From Human Pluripotent Stem Cells Engineered for Detection of Pyrogens
Source: Cell Prolif. 2026 May 21;59(10):e70233. doi: 10.1111/cpr.70233 (PMC13325647; doi:10.1111/cpr.70233)
Supplement: Supplementary file 2 [file CPR-59-e70233-s002.docx]

**Supplementary Figure 1. hESC-Mono exhibit comparable marker expression profiles to PBMC-Mono, distinct from those of THP-1 cells**
(A) Geometric mean fluorescence intensity (gMFI) of CD14 and CD11B in PBMC-Mono (top), hESC-Mono (middle), and THP-1 (bottom) (n ≥ 3, mean ± SD). Bars represent the gMFI of the indicated markers (blue) compared to the unstained control (gray).

(B) Geometric mean fluorescence intensity (gMFI) of CD80 and CD206 in PBMC-Mono (top), hESC-Mono (middle), and THP-1 (bottom) (n = 3, mean ± SD). Bars represent the gMFI of the indicated markers (colored) compared to the unstained control (gray).

**Supplementary Figure 2. Confirmation of NF-κB-Luc reporter integration site**
(A) Genomic validation confirms precise NF-κB-Luc integration at 5′/3′ homology regions in hESC clones (Dashed lines indicate cropped areas, Ctrl: parental hESCs as control).

**Supplementary Figure 3.** **Determination of optimal assay conditions for NF-κB-Luc hESC-Mono response to non-endotoxin stimuli**
(A-B) Luciferase activity in NF-κB-Luc hESC-Mono, following 6-hour stimulation with 10 μM Resiquimod (R848) (A) or 10 μM Imiquimod (R837) (n=5, mean ± SD).

(C-F) Kinetics of NF-κB activation across varying cell densities (1×10^4^, 5×10^4^, 1×10^5^, 2×10^5^ cells/well) following LTA (C) or Zymosan (E) exposure (0-10 ng/mL, 6 hours). Bar graphs represent luciferase activity (n=3, mean ± SD).

Time-course profiling. Concentration-dependent NF-κB activation by LTA (D) or Zymosan (F) (0-10 ng/mL) in 1×10⁵ cells/well across 6–48 hours of post-stimulation. Bar graphs represent luciferase activity (n=3, mean ± SD).

Data are representative of at least two independent experiments. Error bars represent mean ± SD. Statistical significance was assessed using one-way ANOVA. *, *P* < 0.05; **, *P* < 0.01; ***, *P* < 0.001; ****, *P* < 0.0001; NS denotes not significant.
